# Recurrent Catatonia and Demyelinating Disorders

**DOI:** 10.7759/cureus.41656

**Published:** 2023-07-10

**Authors:** Stephanie M Jiang, Marybeth Koepsell, Bhargav Patel, Argyro Athanasiadi

**Affiliations:** 1 Department of Psychiatry and Health Behavior, Augusta University Medical College of Georgia, Augusta, USA

**Keywords:** schizoaffective disorder, electroconvulsive therapy (ect), inflammatory and demyelinating disease, recurrent catatonia, catatonia

## Abstract

Catatonia, a neuropsychiatric syndrome characterized by psychomotor and behavioral symptoms, can be associated with various underlying conditions, including demyelinating diseases such as multiple sclerosis. This paper presents a case study of a 47-year-old female with recurrent catatonic relapses and an underlying demyelinating disease. The patient exhibited symptoms of confusion, decreased oral intake, and difficulty with movement and speech. Neurological examinations, brain imaging, and laboratory tests were conducted to evaluate the etiology and guide treatment. The patient showed improvement with lorazepam and electroconvulsive therapy (ECT). However, relapses occurred after the abrupt withdrawal of medication. The case study highlights the potential connection between demyelinating diseases and catatonia and emphasizes the importance of considering demyelinating diseases in the workup, treatment, and relapse prevention of catatonia. Further research is needed to explore the mechanisms underlying the relationship between demyelination and catatonia and to investigate how different etiologies may impact the recurrence rates of catatonic episodes.

## Introduction

Catatonia is a potentially life-threatening neuropsychiatric syndrome first introduced in 1874 [1]. Once thought to be a subtype of schizophrenia, catatonia is now recognized by the Diagnostic and Statistical Manual of Mental Disorders, Fifth Edition (DSM-5) as the presence of three or more psychomotor or behavioral symptoms, such as catalepsy, wavy flexibility, mutism, echolalia, and echopraxia [2,3]. Catatonia can occur due to underlying conditions other than schizophrenia and has been identified in patients with multifactorial medical and psychiatric disorders [4]. By percentages, the number one condition associated with catatonia is bipolar disorder, with an estimated 43% of cases, and schizophrenia comes in second at 30% of cases [5].

Other associated disorders range across systems and include psychiatric and neurological syndromes such as obsessive-compulsive disorder, post-traumatic stress disorder, alcohol withdrawal, benzodiazepine withdrawal, tic disorders, dystonia, and various forms of encephalitis such as HIV encephalopathy [4,5]. Various bacterial, viral, and parasitic infections of the CNS system can also present with catatonia. In addition to infections, the immune system and autoimmune diseases can also trigger a catatonic episode, though the mechanism is unclear. It appears that the most common of the autoimmune triggers is N-methyl- D -aspartate receptor (NMDA) receptor encephalitis [6]. Since the initial onset of the COVID-19 pandemic, there have also been case reports of catatonia associated with COVID-19 [7]. With such a wide variety of etiologies, the mechanisms for how they induce catatonia and the mechanisms of catatonia itself are still uncertain, though abnormalities of neurotransmission (especially GABA, glutamate, and dopamine), cerebral blood flow, changes in the functioning of the supplementary motor cortex, and changes in gamma-aminobutyric acid (GABA) receptor density have all been implicated [8].

Relevant to this case, there is evidence to suggest that catatonia may occur more frequently with demyelinating diseases, although the exact nature of this relationship is not well understood [9,10]. A possible hypothesis is that abnormalities in myelin genes and the proteins they encode for can trigger inflammation of myelinated neural tracts, leading to demyelination, axonal damage and loss, and signs of catatonia [10]. A similar animal study showed proof-of-principle that loss-of-function genotypes of CNS myelin-associated genes such as CNP combined with an inflammatory environment can lead to a "catatonia depression" syndrome [11]. The importance of this case is twofold, as it (a) gives further evidence of the connection between demyelinating diseases and catatonia and (b) highlights considerations for workup, treatment, and relapse prevention in catatonia in these cases. Treatment of catatonia typically involves varying doses of lorazepam and/or electroconvulsive therapy (ECT) [3,12]. Here, we present the case of a patient with recurrent catatonic relapses and an underlying demyelinating disease.

## Case presentation

The patient is a 47-year-old female with no significant medical conditions and a psychiatric history of schizoaffective disorder, bipolar type. Her home medications include aripiprazole, topiramate, and lithium. She is a single mother who works as a massage therapist. She lives at home with her parents, who help manage her medications. She has a long history of psychotic and manic episodes, a catatonic episode one year prior, and suicidal ideation that required hospitalization and inpatient psychiatric care due to a combination of family/life stressors and nonadherence to medications. Family history is significant for multiple sclerosis.

The patient first presented to our ED (July 2022) for concerns about confusion, decreased oral intake, and difficulty speaking and moving her legs. Her parents noted the patient had symptoms of malaise, nausea, and abdominal pain that began four days prior. On evaluation in the ED, the patient was afebrile, and vitals were notable for tachycardia in the 140s. The patient was nonverbal and unresponsive to commands. Her Busch-Francis Catatonia Scale (BFCS) was positive for immobility/stupor, mutism, rigidity, withdrawal, and autonomic abnormality for a total score of 10. The physical exam was significant for rigidity, 3+ deep tendon reflexes, and the Hoffman sign in the bilateral upper extremities.

Initial lab workup for altered mental status and suspected catatonia included a complete blood count, comprehensive metabolic panel, lipid panel, urinalysis, urine toxicology screen, salicylate level, thyroxine and thyroid-stimulating hormone (TSH) levels, CK, troponin, chest X-ray, EKG, and head CT. The patient was found to have an elevated white blood cell count (13.9 thous/mm^3^), elevated CK (513), elevated troponin (0.29 ng/mL), and subtherapeutic lithium levels (0.5 mEq/L). The EKG showed QTC prolongation. The CT head was negative for intracranial pathology. T4/TSH was normal. The urine dipstick test was negative. She remained unresponsive to commands, with limited eye contact and participation in the interview. She had mild bilateral extremity rigidity and withdrew in pain. Due to a lorazepam shortage, the patient was started on IV Diazepam (5 mg Q8hr on day 1, 10 mg Q8hr on day 2, and 20 mg Q8hr on day 4). By the seventh day, the patient had minimal improvement following the trial of IV benzodiazepines, prompting suspicion of other contributing etiologies in addition to catatonia, such as encephalopathy.

**Figure 1:**
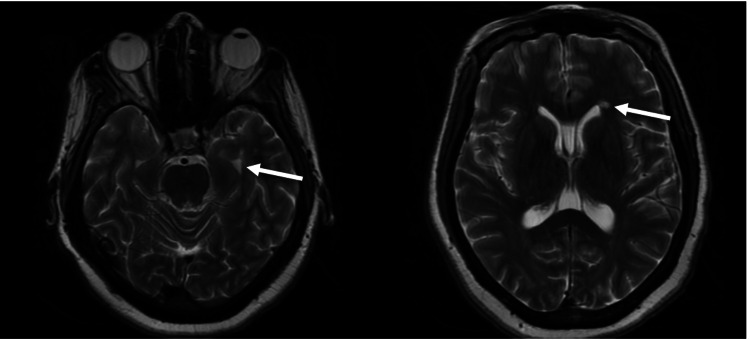
T2-weighted magnetic resonance images of the head. Horizontal cuts show numerous periventricular white matter hyperintensities with involvement of the temporal horns, especially the left, giving evidence of demyelinating lesions.

Neurology was consulted for the evaluation of encephalopathy, clonus, and spasticity, with minimal improvement on benzodiazepines. The neurologic workup included MRI, LP, and EEG. MRI w/o contrast showed abnormal T2 flair hyperintensities involving the cingulate gyri and possibly the left mammillary body, insula, and mesial temporal lobes (left greater than right), which was concerning for viral or limbic encephalitis or seizure sequela. MRI also showed numerous periventricular white matter T2 flair hyperintensities with involvement of the temporal horns. Repeat MRI of the brain with and without contrast showed a T1 hypointense signal involving the previously seen areas of T2/Flair hyperintense signals with a small area of delayed enhancement along the ependymal surface of the occipital horn of the left lateral ventricle (Figure 1). The EEG showed diffuse encephalopathy with no seizure activity.

At this point in time, the differential remained broad and included multiple sclerosis, autoimmune encephalitis, and acute disseminated encephalomyelitis. Viral encephalitis was deemed less likely after the patient showed no improvement with acyclovir. CSF antibodies were negative for anti-GAD and anti-NMDA receptor antibodies, which helped exclude stiff-person syndrome and autoimmune encephalitis. There was a concern for paraneoplastic encephalitis, though CT of the abdomen and pelvis was negative. Since there was low suspicion of organic brain pathology, catatonia was the leading diagnosis. After three days of methylprednisolone, the patient was able to speak, and her rigidity slightly improved. The following day, however, she appeared paranoid and scared, with incomprehensible speech. She continued to have hypoactive movements, resistance to being touched, and significant negativism. Her affect was labile and intense, with loud, pressured, and dysarthric speech. Her insight and judgment were poor. We were unable to obtain a complete mental status exam due to her condition. ECT treatment was started due to her declining condition. After the fifth ECT treatment, her Bush-Francis score was 18. After the eighth treatment, her Bush-Francis score was 8, and she had improved rigidity, speech fluency, stable effect, and a linear thought process. She also became alert and oriented to people and places. After her 11th and final ECT treatment, the patient no longer displayed posturing and was ambulating without issue. She was able to fully participate in interviews, and her mental status exam was negative for psychomotor, mood, or cognitive findings. The patient was discharged on a dosage of aripiprazole (15 mg QD) and lithium (600 mg QHS).

**Figure 2:**
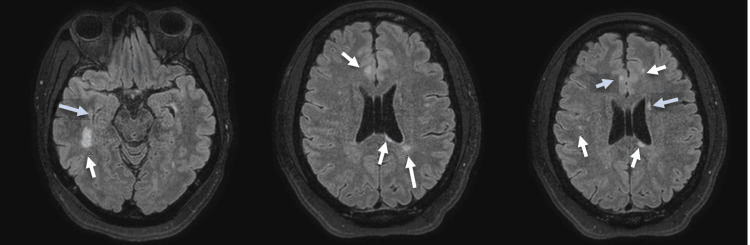
FLAIR magnetic resonance images of the head. Multifocal evolving FLAIR signal abnormality lesions over time when compared to previous MRIs five months ago with interval enhancement of the subependymal lesion in the posterior superior margin of the body of the left lateral ventricle at its junction with the left ventricular atrium, concerning for demyelinating disease such as seen in multiple sclerosis.

Five months later (January 2023), the patient was admitted to the hospital with a similar presentation: nausea, abdominal pain, and generalized weakness, followed by decreased oral intake, confusion, and slowed movements. Per her mother, she had been off lithium for three months due to lethargy but had continued taking Abilify daily. Per her parents, whom she lived with, she was doing well and following up with her outpatient appointments and performing regular activities, and they had no reason for concern up until a week before her hospitalization. On exam, her BFCS was 12, and her lithium levels were undetectable. A brain MRI showed a new enhancing periventricular lesion, consistent with nonspecific white matter lesions from her prior visit in July 2022 (Figure 2). A neurological exam showed lower extremity spasticity but no focal deficits. LP was positive for oligoclonal bands but negative for pleocytosis. Neurology diagnosed her with a nonspecific demyelinating process and treated her for three days with methylprednisolone. They had concerns about MS, so they scheduled the patient in their outpatient MS clinic for a post-discharge evaluation. The patient responded well to lorazepam, but it is unclear whether the concurrent use of methylprednisolone contributed to the rapid recovery. During her six-day hospital stay, she improved without needing ECT treatments. On the day of discharge, she had no signs of catatonia but still exhibited a depressed mood and slowed speech. She was discharged on lorazepam, aripiprazole, and lithium, with plans to taper lorazepam across seven weeks to be followed up with outpatient psych due to a recurrence of her catatonia. For the demyelinating disease, the patient was also scheduled to see our outpatient neurology clinic.

The next week, the patient was admitted to the hospital due to a relapse of her catatonia due to an abrupt change in her medication regimen by her outpatient provider. Per the patient’s mother, her speech had declined since being discharged from the hospital after lithium was discontinued and lorazepam was abruptly decreased from 2 mg TID to 0.5 mg nightly by her outpatient provider. Soon, the patient stopped drinking and talking. On admission, her BFCS was 17. The patient was restarted on lorazepam and lithium. Due to minimal improvement, the patient had a total of five ECT treatments before she improved back to baseline. The patient was discharged on lorazepam without taper, aripiprazole, and lithium.

## Discussion

This case highlights a patient with a concurrent demyelinating disorder and catatonia. Our patient is unique because there are few cases in the literature that have reported patients with demyelinating disorders and recurrent catatonia [9,13,14].

One case report from 1999 details a 29-year-old male patient hospitalized with depression with psychotic features and catatonic symptoms of stupor, mutism, immobility, and rigidity. His catatonic symptoms resolved following a spontaneous generalized seizure, similar to one induced by electroconvulsive therapy [15]. He also reported neurological symptoms such as intermittent weakness in his extremities and face. After a detailed cerebrospinal fluid analysis of elevated immunoglobulins and oligoclonal bands and an MRI showing evidence of white matter hyperintensities, he was discharged with a diagnosis of multiple sclerosis with secondary catatonic disorder. Over the next two years, the patient experienced one recurrence of his catatonia due to a change in his medication regimen that involved mutism and immobility [9]. Another case report by Pontikes and Dinwiddie describes a 28-year-old male patient with preexisting diagnoses of multiple sclerosis and depression. He was admitted to the hospital after developing signs of mutism, immobility, posturing, and staring. After not responding to a trial of lorazepam, the patient received 4 total ECT treatments over 8 days until his symptoms resolved. However, six weeks after discharge, the patient developed recurrent signs of catatonia, including paucity of speech and immobility. He received 23 additional ECT treatments over the next 15 months [13]. Another case report describes a 16-year-old female with epilepsy and neuromyelitis optica (NMO) who was brought to the emergency department with symptoms of catatonia and psychosis and scored 11 on the BFCS. She was treated with lorazepam for her catatonia in addition to risperidone for her psychosis. She was discharged after five weeks of hospitalization with residual mild cognitive impairment [14]. It is unknown whether she had relapses.

Although our patient did not have a diagnosis of multiple sclerosis or NMO, her MRI results after both her first and second admissions at our hospital for catatonia-like symptoms showed evidence of demyelinating disease and new demyelinating lesions. A mouse study on the myelin-associated gene CNP, found in both mice and humans, showed how the reduced expression of the gene led to a catatonia-depression syndrome with symptoms of bizarre posturing and social withdrawal. While the exact mechanism is unknown, it appears to be a "two-hit" hypothesis, as the mouse study showed that the effects only surfaced in late life in conjunction with a second "pro-inflammatory hit" found upon aging [11]. In humans, the AA genotype of the same gene at SNP rs2070106 has been found to have reduced expression in patients with severe mental illnesses like schizophrenia in postmortem studies, as well as evidence of axonal loss on diffusion tensor imaging in the frontal corpus callosum [11]. The authors go on to conclude that changes in subcortical white matter could be the cause of complex neuropsychiatric syndromes rather than simply the effect. Another study performed on mice with the same CNP variant showed that microglia ablation alleviated the symptoms of catatonia in CNP mutants [10]. This further elucidates both an association between catatonic symptoms and demyelinating disorders and potential avenues for treatment. There is further evidence of dysregulation of the immune system and its association with catatonia in autoimmune demyelinating disorders [6].

As evidenced by our patients and those with existing MS or NMO, different etiologies of catatonia, as well as withdrawal of medications, may increase or decrease the likelihood of recurrence [9,13,14]. Many case reports have also been published describing catatonia two to seven days after benzodiazepines were rapidly tapered or withdrawn [16]. However, elderly patients or patients with underlying medical conditions are more susceptible to benzodiazepine withdrawal catatonia [17]. Our patient initially responded to lorazepam and ECT, but she relapsed multiple times following lorazepam withdrawal or mild adjustments to her psychiatric medications. She relapsed when her lorazepam was decreased only for one day and then appeared to be refractory to initial treatment again with lorazepam, which is unlikely for patients without underlying medical conditions. Our case, as well as the literature above, shows that the first-line treatments lorazepam and/or ECT are effective when the catatonia is secondary to demyelinating disease, but there is a potentially higher risk of relapses and resistance to treatment, and treatment for the demyelinating disease should also be considered concurrently [18,19,20].

## Conclusions

Ultimately, regardless of etiology, treatments for catatonia currently appear to be the same, including increasing doses of lorazepam as well as electroconvulsive therapy. Electroconvulsive therapy is also becoming an option for treating psychosis in patients with multiple sclerosis. Additionally, addressing underlying causes such as bipolar disease or schizophrenia in patients with demyelinating disorders, as well as treating the demyelinating disorders, can decrease the likelihood of relapse into catatonia. Our patient highlights the importance of recognizing the possibility of the development of catatonia early in patients with demyelinating disorders, as well as suspecting underlying demyelinating disorders in patients with catatonia symptoms that also exhibit abnormal neurological symptoms. Further research is necessary regarding the relationship between myelination and catatonia and how different etiologies for catatonia may impact rates of recurrence.
